# Variation of Corrosion Characteristics and Tensile Performances of WE43 Alloy Under Marine Atmospheric Environment

**DOI:** 10.3390/ma17215353

**Published:** 2024-11-01

**Authors:** Lin Xiang, Fei Li, Xinrui Wu, Feiyue Zhang, Jianquan Tao, Maochuan Wang, Wei Lei, Xudong Ran, Hui Wang

**Affiliations:** Southwest Technology and Engineering Research Institute, Chongqing 400039, China; xlin0731@163.com (L.X.); 18523727206@163.com (F.L.); 310227614@163.com (X.W.); 18723003055@163.com (F.Z.); bruce_xlin@163.com (M.W.); autismpait@gmail.com (W.L.); xudongran0828@outlook.com (X.R.); cq15825950152@163.com (H.W.)

**Keywords:** WE43 alloy, marine environment, corrosion, microstructure, tensile properties

## Abstract

This study aims to examine the variation in corrosion characteristics and tensile properties of WE43 magnesium alloy in an actual marine atmospheric environment by means of outdoor exposure tests. The macroscopic corrosion morphology, microstructure, and tensile properties were analyzed. The results indicated that WE43 alloy will corrode rapidly during exposure under marine atmospheric environmental conditions, resulting in a loose and porous Mg(OH)_2_ layer on the surface. The Mg matrix was mainly consumed as an anode, leading to the occurrence of corrosion pits. With the increase in exposure time, both the tensile strength and plasticity of WE43 alloy gradually deteriorated. After exposure for six months, the elongation and area reduction were significantly reduced, with a reduction ratio of more than 50%. After 18 months of exposure, the ultimate strength of the alloy decreased from 359 MPa to 300 MPa. According to an analysis of fractures in the alloy, the corrosion pits on the sample surface were the main reason for the decrease in tensile properties.

## 1. Introduction

With the rapid development of the aerospace industry, the weight reduction of structural components has become a huge challenge [1]. Magnesium and its alloys have been considered popular engineering structural materials in aerospace, transportation, and electronic technologies because of their high strength, low density, and thermal conductivity [2,3,4,5,6,7,8,9]. Thus, many researchers have focused on the effects of heat treatment, deformation parameters, and various chemical elements on the microstructure and mechanical properties of magnesium alloys [10,11,12]. However, Mg alloys possess poor corrosion resistance [13,14]. Therefore, when Mg alloy components operate in corrosive media, especially in marine environments, they will undergo corrosion leading to the degradation of mechanical properties.

Up to now, many studies have explored the corrosion behavior of magnesium alloys [15,16,17,18]. For instance, Li et al. studied the corrosion behavior of WE43 and AZ80 alloys in NaCl and Na_2_SO_4_ solutions, and found that when immersed in Na_2_SO_4_ solution, WE43 alloy shows a unique micro-galvanic corrosion behavior [15]. Liu et al. observed the corrosion performance of extruded and forged Mg-Gd-Y-Nd-Zr alloys in 0.6 M NaCl solution, indicating that the extruded alloy showed uniform corrosion, while the forged alloy suffered severe local galvanic corrosion [16]. However, most studies have been carried out in simulated corrosive environments, which cannot reflect the actual corrosion behavior of Mg alloys in the natural environment. Therefore, this study aimed to investigate the variation in microstructure and tensile properties of WE43 magnesium alloy in an actual marine atmosphere environment via outdoor exposure tests. After outdoor exposure for different lengths of time, the macromorphology, cross-sectional morphology, microstructure, and tensile properties of WE43 alloy were analyzed.

## 2. Experimental

**Figure 1:**
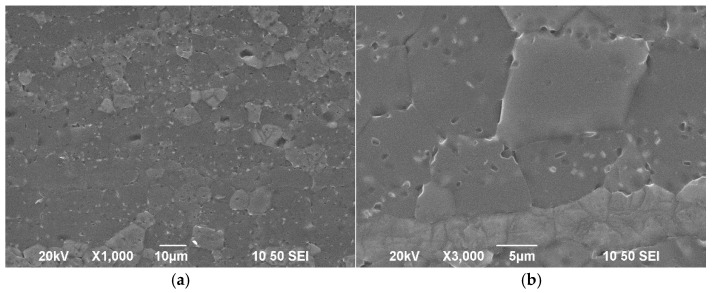
Initial microstructure of WE43 alloy under (**a**) low magnification and (**b**) high magnification.

**Figure 2:**
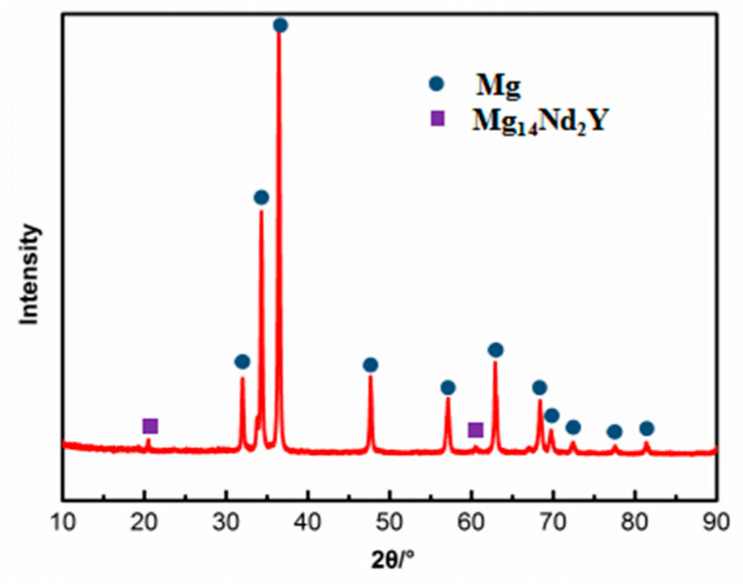
XRD patterns of WE43 alloy.

Rolled WE43 that had undergone T5 heat treatment was used in this study. Its chemical composition is shown in Table 1, and the initial microstructure is shown in Figure 1. As observed, the present alloy consists of α-Mg matrix and precipitates, of which the grains are typically fine. Meanwhile, the α-Mg matrix is equiaxed, and precipitates are mainly distributed in the matrix, while a minority of the precipitates are located at the grain boundaries. Figure 2 shows the XRD patterns of WE43 alloy. It is obvious that the phases of WE43 alloy comprise an α-Mg matrix and a β(Mg_14_Nd_2_Y) phase. Therefore, it can be concluded that the precipitates in WE43 alloy were β(Mg_14_Nd_2_Y).

Plate-shaped samples of 100 mm × 50 mm × 3 mm and dumbbell-shaped samples with a dimension of 10 mm in diameter were cut from a WE43 rolled plate and placed in an outdoor exposure field (300 m from the coastline) in Wanning, Hainan Province. The test field possessed a typical hot and humid marine atmospheric environment. All samples were firmly installed on the exposure test rack and inclined at a 45° angle from the horizontal surface. After exposure intervals of 6, 12, and 18 months, one piece of plate sample and three pieces of dumbbell-shaped samples were picked for organizational observation and mechanical performance testing, respectively. The microstructures were observed by optical microscopy (OM, Leica DFC320, Wetzlar, Germany) and scanning electron microscopy (SEM, Jeol JSM-6390A, Tokyo, Japan). The second phase composition was analyzed using the D/MAX 2200 PC X-ray diffraction (Rigaku Corporation, Tokyo, Japan) instrument and a transmitted electron microscope (TEM, TECNAI F30 G2, Hillsboro, OR, USA). An MTS E45.105 servo-hydraulic tensile machine was used to test the tensile performance at room temperature and tensile speed of 2 mm/min. The average value of three test results is shown in this study. The weight loss was analyzed according to the Chinese Standard 16545-2015 [19] (Corrosion of metals and alloys—Removal of corrosion products from corrosion test specimens).

## 3. Results and Discussion

### 3.1. Macroscopic Corrosion Morphology

**Figure 3:**
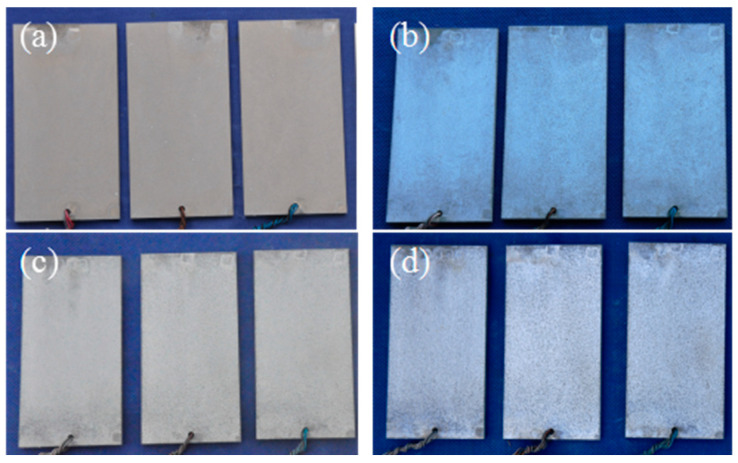
Macroscopic morphology of WE43 alloy exposed for different lengths of time: (**a**) 1 month, (**b**) 6 months, (**c**) 12 months, (**d**) 18 months.

Figure 3 shows the macroscopic morphology of WE43 alloy after different lengths of exposure time in a hot and humid marine atmospheric environment. It is obvious that the WE43 alloy corroded quickly, as its surface turned dark gray after one month of outdoor exposure, as shown in Figure 3a. As the exposure time increased to six months, the sample surface changed from dark gray to gray and white, as shown in Figure 3b. When the exposure time increased to 12 months, the sample surface changed to dark gray again, as shown in Figure 3c. After further extending the exposure time, the color of the sample surface changed again, as shown in Figure 3d. That is, as the exposure time is extended, the sample surface shows a phenomenon of alternating dark gray and gray and white color change. Table 2 shows the weight loss data of the WE43 alloy after exposure times of different length in a hot and humid marine atmospheric environment. The analysis of the average weight loss showed that the values after exposure for 6 months, 12 months and 18 months were 0.2544 g, 0.4283 g, and 0.6177 g, respectively. Correspondingly, the corrosion rate of the present alloy was 0.128 g/(m^2^ d), 0.1075 g/(m^2^ d), and 0.1032 g/(m^2^ d), respectively. This indicates that the specimens’ weight loss increases with the increase in exposure time. Compared with aluminum alloys [20,21], WE43 alloy corroded more quickly.

**Figure 4:**
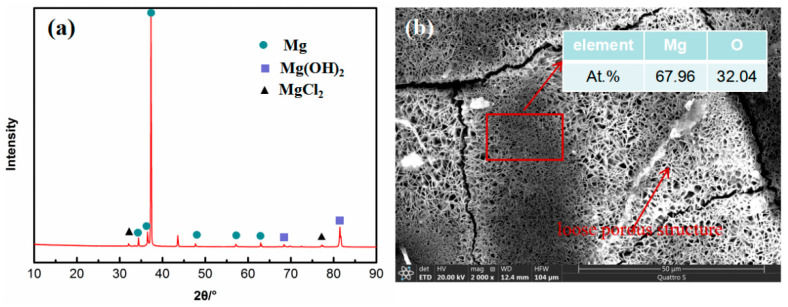
(**a**) XRD patterns and (**b**) the morphology of corrosion product on surface of WE43 alloy after outdoor exposure.

XRD was used to analyze the surfaces of the samples after outdoor exposure under hot and humid marine atmospheric environmental conditions, as shown in Figure 4a. As indicated, the main corrosion products on the sample surface are Mg(OH)_2_ and MgCl_2_. It has been demonstrated that Mg alloys are extremely prone to corrosion in humid environments [22,23]. According to literature [24], Mg matrix is relatively active with a standard electrode potential of −2.37 V, which is often consumed as an anode, and producing Mg(OH)_2_ on the sample surface. The reaction formula is as follows [25].

Mg − 2e^−^ → Mg^2+^ (anodic reaction)

2H_2_O + 2e^−^ → 2OH^−^ + H_2_↑ (cathodic reaction)

Mg + 2H_2_O → Mg(OH)_2_↓ + H_2_↑ (overall reaction)

Further analysis of the corrosion product via EDS is shown in Figure 4b. The main elements contained are Mg and O, of which the atomic percentage is about 2:1, further verifying that the corrosion products are Mg(OH)_2_. Since the reaction produces gas, it will destroy the deposited Mg(OH)_2_ surface film during the release of gas and form a loose porous structure, as shown in Figure 4b. Thus, the corrosion products have a weak protective effect on the matrix. According to previous research, the Cl^−^ deposition rate at the Wanning test site can reach about 14.5875 mg/(m^2^ d) [26,27]. Therefore, as the corrosion progresses, Cl ions in the hot and humid marine atmospheric environment easily enter the interior of the Mg alloy through the loose structure and react with the Mg matrix to generate MgCl_2_.

### 3.2. Micro-Corrosion Characteristics

**Figure 5:**
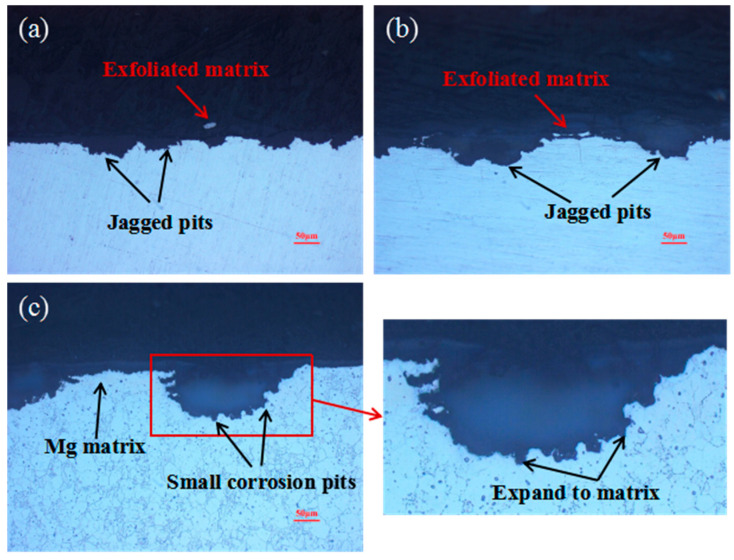
Cross-sectional morphology of WE43 alloy after different lengths of outdoor exposure time: (**a**) 6 month, (**b**) 12 months, (**c**) 18 months.

Figure 5 shows the cross-sectional morphology of WE43 alloy after outdoor exposure for different lengths of time. As indicated, the corrosion characteristics of the alloy are always pitting corrosion, corrosion pits that appear jagged, and corrosion pits that tend to be connected to one another as the corrosion progresses, as shown in Figure 5a. Moreover, the exfoliated matrix can be found by analyzing the corrosion pits and the matrix, as shown in Figure 5c. As observed, after pitting occurs, many small corrosion pits appear at the edges of large corrosion pits, and these mainly exist in the Mg matrix. The small corrosion pits show a tendency to expand into the surrounding matrix, as shown in the magnified image of the red rectangle in Figure 5c. Accordingly, it can be speculated that the surfaces of the samples are in contact with humid air, thereby forming galvanic corrosion conditions and causing Mg matrix corrosion, forming corrosion pits. Since the formed Mg(OH)_2_ corrosion products have a loose porous structure and cannot protect the Mg matrix from the corrosive environment, the corrosion behavior will continue. As a result, the junction between the corrosion pits and the Mg matrix becomes a new location for corrosion, and newly formed small corrosion pits expand around the Mg matrix. After expanding to a certain extent, the adjacent corrosion pits connect to one another, causing the Mg matrix to peel off. Therefore, the macroscopic morphologies of the samples exhibit alternating dark gray and gray-white phenomena with increasing length of exposure time.

**Figure 6:**
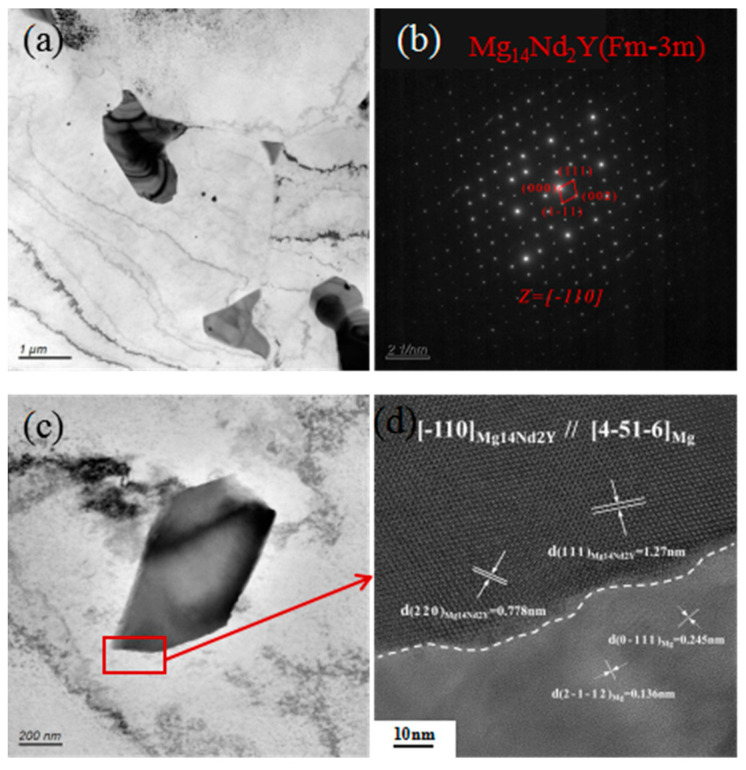
TEM images of WE43: (**a**) bright-field image, (**b**) diffraction spots of the secondary phase, (**c**) morphology of the secondary phase, (**d**) high-resolution images of the secondary phases.

Figure 6 shows the TEM images of WE43 alloy. As shown in Figure 6a, there is a second phase within the Mg matrix and grain boundaries. By diffraction spot analysis of the second phase, the second phase was found to be β(Mg_14_Nd_2_Y) phase, which further verified the XRD results. High-resolution analysis results show that the second phase has a face-centered cubic structure, a = b = c = 2.23 nm, and the Mg matrix has a hexagonal close-packed structure, a = b = 0.32 nm, and c = 0.52 nm. Meanwhile, the β phase and Mg matrix maintain an incoherent relationship, but there is a certain orientation relationship: [-110]_Mg14Nd2Y_∥[4-51-6]_Mg_. Typically, the precipitation of the intergranular second phase can easily lead to intergranular corrosion. However, for WE43 alloy, due to the incoherent relationship between the second phase and the Mg matrix, the distortion energy of the phase interface is low, and stress concentration will not be formed or is weak, so WE43 has no intergranular corrosion. Therefore, under the galvanic corrosion conditions formed by the active Mg matrix and the second phase, the corrosion type present in the WE43 alloy is mainly pitting corrosion, and the Mg matrix is continuously consumed.

**Figure 7:**
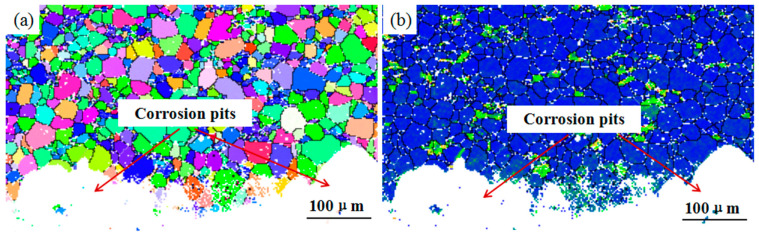
EBSD maps of WE43 alloy after 18 months of outdoor exposure in Wanning: (**a**) IPF map, (**b**) KAM map.

Figure 7 shows the EBSD maps of WE43 after 18 months of outdoor exposure. According to the IPF results, the Mg matrix is a typical recrystallized equiaxed microstructure with fine and uniform grains. Figure 7b illustrates dislocations and lattice distortion near corrosion pits. As demonstrated, the contact position between the corrosion pits and the Mg matrix appears blue, indicating there is no significant dislocation accumulation and stress concentration, and there is no significant stress concentration inside the matrix. This is consistent with TEM images, further demonstrating that the main type of corrosion in WE43 alloy is pitting corrosion, and intergranular corrosion will not occur. Additionally, corrosion pits expand randomly around the Mg matrix during corrosion, because there is no obvious stress concentration inside the WE43 alloy. 

**Figure 8:**
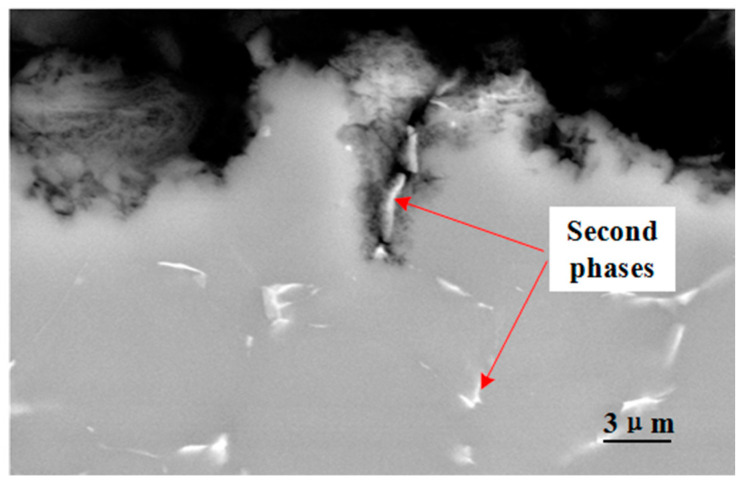
Cross-sectional morphology of the corrosion products.

Figure 8 shows the cross section of the corrosion products. It can be found that corrosion behavior occurs near the white second phase under the hot and humid marine atmospheric environmental conditions. Meanwhile, in the corrosion products layer, the second phase remains. This proves that during the corrosion process, the second phase serves as the cathode and the Mg matrix serves as the anode and is continuously consumed.

### 3.3. The Degradation of Mechanical Property

**Figure 9:**
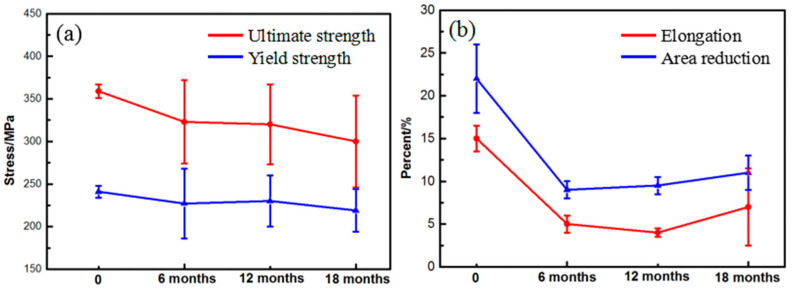
Variation in tensile performance of WE43 alloy after different lengths of outdoor exposure time: (**a**) Strength, (**b**) plasticity.

Figure 9 shows the variation in tensile performances of WE43 alloy after outdoor exposure times of different length under hot and humid marine atmospheric environmental conditions. It is obvious that the strength and plasticity of WE43 alloy have undergone significant changes. As shown in Figure 9a, as the exposure time increases, the tensile strength and yield strength continue to decrease. After an 18-month exposure, the ultimate strength of WE43 decreased from 359 MPa to 300 MPa. As shown in Figure 9b, after a 6-month exposure, the elongation and area reduction of WE43 alloy decreased by more than 50%. According to the results of these corrosion characteristics, the alloy would corrode quickly in a humid environment, forming jagged corrosion pits on the sample surface. When stretching, the tips of corrosion pits face the inside of the sample and are perpendicular to the direction of stress, which can easily become the location for crack initiation, resulting in a decrease in the performance of WE43. However, after 6 months of exposure, if the exposure time continues to be extended, the elongation and area reduction of WE43 alloy do not continue to show a decreasing trend, indicating that the impact of corrosion behavior on the plasticity of WE43 is mainly concentrated in the early stage of corrosion.

**Figure 10:**
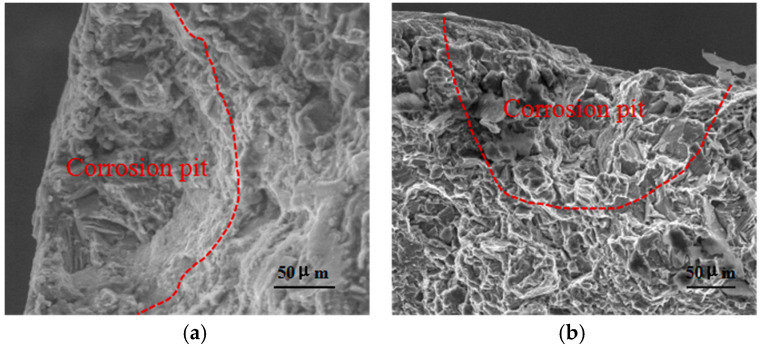
Fracture morphology of WE43 alloy after outdoor exposure under hot and humid marine atmospheric environment conditions: (**a**) 6 months, (**b**) 12 months.

Figure 10 shows the fracture morphology of WE43 alloy after outdoor exposure under hot and humid marine atmospheric environmental conditions. As indicated, the fracture includes two morphologies: One is the dimple-free fracture near the corrosion pits, and the second is the conventional fracture morphology outside the corrosion pits. The above phenomena show that during the stretching process, corrosion pits become the location of crack initiation and the weak points involved in WE43 cracking, while locations other than corrosion pits maintain the original fracture mode and form a large number of dimples, which also explains why the plasticity of WE43 does not decrease with exposure time.

## 4. Conclusions

The corrosion characteristics and mechanical property changes of WE43 in a hot and humid marine atmospheric environment were investigated. The main conclusions are as follows:

(1) In a hot and humid marine atmospheric environment, WE43 alloy corroded rapidly, forming a loose and porous Mg(OH)_2_ layer on the surface, which had a weak protective effect on the matrix. Thus, as the corrosion progressed, in a hot and humid marine atmospheric environment Cl ions easily entered the Mg alloy through the loose structure and reacted with the Mg matrix to generate MgCl_2_. As a result, the weight loss of WE43 alloy increased gradually.
(2) The main corrosion mechanism seen in WE43 alloy was pitting corrosion, which formed jagged corrosion pits. As the corrosion progressed, the Mg matrix was continuously consumed as an anode, and the corrosion pits expanded into the surroundings. After expanding to a certain extent, adjacent corrosion pits were connected to each other, resulting in the Mg matrix peeling off.
(3) The formation of corrosion pits promoted the initiation of cracks, which influenced the tensile properties of the alloy significantly. After only 6 months of exposure, the elongation and area reduction of WE43 alloy were significantly reduced, with a reduction ratio of more than 50%. And after an 18-month exposure, the ultimate strength decreased from 359 MPa to 300 MPa.
(4) According to the results of this study, we think that some protective methods should be employed to prolong the service properties of WE43 alloy components when they are used under marine atmospheric environmental conditions.

## Figures and Tables

**Table 1:** Chemical compositions of treated WE43 (wt.%).

Element	Nd	Gd	Y	Zr	Zn	Li	Fe
Content/%	2.01	0.74	3.96	0.50	<0.01	<0.01	<0.01

**Table 2:** The weight loss data of WE43 alloy after different lengths of exposure time.

Samples No.	Exposure Time	Weight Loss/g	Average of Weight Loss/g	Average of Corrosion Rate/(g/(m^2^ d))
1	6 months	0.3146	0.2544	0.128
2		0.2557		
3		0.1929		
4	12 months	0.4294	0.4283	0.1075
5		0.4173		
6		0.4384		
7	18 months	0.6195	0.6177	0.1032
8		0.5985		
9		0.6350		

## Data Availability

The original contributions presented in the study are included in the article, further inquiries can be directed to the corresponding author.
